# Development of multidose thermotolerant formulations of a vector-based Covid-19 vaccine candidate, NDV-HXP-S in different product formats: Stability and preservative efficacy study

**DOI:** 10.1016/j.jvacx.2024.100535

**Published:** 2024-07-27

**Authors:** Anan Bzami, Changcheng Zhu, Marcus Estrada, Jessica A. White, Manjari Lal

**Affiliations:** PATH, 2201 Westlake Avenue, Suite 200, Seattle, WA 98121 USA

**Keywords:** SARS-CoV-2, Vaccine multidose, Stability, Newcastle Disease Virus, Thermotolerant, Lyophilization, NDV-HXP-S, Preservative

## Abstract

Current lead coronavirus vaccines require continuous cold or ultra-cold storage from the manufacturing site to the field to maintain protective efficacy. Since cold chain capacity is limited and complex, logistics planning is crucial to limit vaccine wastage.[1] The restrictive storage concerns also make it difficult to share vaccines between public health departments and neighboring states, leading to increased vaccine wastage.[2] A Newcastle Disease Virus (NDV) vector-based severe acute respiratory syndrome coronavirus 2 (SARS CoV-2) vaccine candidate, NDV-HXP-S, offers a cost-effective alternative which aims to improve global access to SARS CoV-2 vaccines.[3] The NDV-HXP-S vaccine candidate can be mass-produced in chicken eggs and has demonstrated efficacy in preclinical studies, as well as acceptable safety and potent immunogenicity in clinical studies.[3,4–10] To further advance the NDV-HXP-S vaccine candidate, this manuscript describes work focused on the development of multidose thermotolerant vaccine formulations (i.e., those which would not require continuous extended refrigeration), making it convenient to use and store, and simplifying transport and distribution logistics, especially in outbreak settings. Liquid and lyophilized formulations for parenteral administration were rigorously screened for the vaccine formulation’s ability to maintain S-antigen stability after exposure to temperature stress at 40 °C, 25 °C, and 2 °C to 8 °C storage for six months. Preservative efficacy was evaluated to enable a multidose liquid vaccine format as well as endotoxin testing in lyophilized formulations. Lead liquid vaccine formations were identified that were able to maintain S-antigen content at 2 °C to 8 °C and 25 °C storage for the entire six-month study. Lead lyophilized vaccine formulations were identified which were able to maintain S-antigen content for six months at 2 °C to 8 °C, 25 °C, and 40 °C. Both the liquid and lyophilized formulations identified are improved thermotolerant SARS-CoV-2 vaccine formulations.

## Highlights

• Liquid and lyophilized formulations-maintained antigen content for 6 months at 25 °C.
• The lyophilized format of the formulation was thermotolerant at 40 °C for 6 months.
• 2PE preservative in the multidose liquid formulation was ineffective in USP51 testing.

## Introduction

Access to SARS-CoV-2 vaccines continues to be a challenge in many low- and middle-income country (LMIC) settings due to manufacturing costs and requirements for cold storage. The viral vector NDV-HXP-S SARS-CoV-2 vaccine candidate employs egg-based vaccine production of a vector-based coronavirus vaccine which enables LMIC vaccine manufacturers to produce a SARS-CoV-2 vaccine. [5], [6], [11] The NDV-HXP-S SARS-CoV-2 vaccine candidate has been shown to be safe and immunogenic in human clinical studies, and Phase 2 and 3 clinical studies and this vaccine candidate has received emergency use licensure in Thailand. [4], [5], [6] As part of the development of this vaccine candidate, the studies described here were conducted to evaluate vaccine formulations for improved thermostability, compatibility of selected formulations with a preservative which allows for multidose vaccine presentations. Previous studies have evaluated the pilot stability of the vaccine candidate (prior to these vaccine formulation studies) and described the stability-indicating S-antigen potency method, which will be used here to identify lead formulations. [7].

This manuscript summarizes the work conducted to successfully identify multiple thermotolerant vaccine formulations, liquid and lyophilized, to further the development of the NDV-HXP-S vaccine candidate. Thermotolerant formulations of NDV-HXP-S were developed for liquid and lyophilized formats for use in parenteral administration. Developed formulations were evaluated on ability to maintain S-antigen stability at 40 °C, 25 °C, and 2 °C to 8 °C over a six-month study period. Lead liquid formulations were identified based on the combined assessments of visual appearance, pH, and S-antigen content (using S-antigen specific ELISA). In addition to the evaluation criteria for the liquid formulations, the lead lyophilized formulations were also evaluated for cake appearance, moisture content, and reconstitution time. To enable future development of a multidose vaccine format, preservative effectiveness testing of the lead liquid formulations was conducted according to USP 51. [12] Similarly, lead lyophilized formulations were subjected to endotoxin testing according to USP 85. This study identified a lead liquid NDV-HXP-S vaccine formulation which maintained S-antigen content and stability at 2 °C to 8 °C for the entire six-month study period. This work also identified a lead lyophilized vaccine formulation which maintained S-antigen content and stability at 40 °C for the six-month study period. Future development of the NDV-HXP-S vaccine candidate in a multidose thermotolerant format will enable wider access to this vaccine and reduce vaccine wastage.

## Materials and methods

### Characterization assays

S-antigen ELISA: All testing and screening used an inhibition ELISA as described in previously published methods to quantify the amount of S-antigen present in NDV-HXP-S formulations. [7] To summarize, test samples and controls are incubated overnight in a deep-well containing a mouse monoclonal (Sino Biological cat# 40592-MM57) that is specific to the S-antigen of the Wuhan Variant of SARS CoV-2. Supernatant is then transferred to a 96 well plate coated with S-antigen to measure any unbound antigen to indirectly measure potency.

Moisture content: Moisture was measured using a Mettler Toledo C20 Karl Fischer Coulometer Titrator. Lyophilization samples were weighed and distributed into anhydrous methanol. Through measuring the moisture of methanol with and without samples, moisture amount was determined by subtracting the moisture from methanol. Moisture content (percentage) was easily calculated by dividing the sample weight times 100.

pH measurements: pH was tested for each formulation using the METTLER TOLEDO™ Micro pH meter for both liquid and lyophilized formulations.

SDS-PAGE: Test samples were evaluated for S-antigen by SDS-PAGE after preparing with 1X sample buffer and heated at 90 °C for five minutes. For each sample, HexaPro S-antigen control (kindly provided by Mt. Sinai Ichan School of Medicine) and protein ladder (Thermo Scientific PageRuler Plus Pre-stained protein ladder) was loaded into a 15-well premade gel and run at 200 V on a bio rad gel running apparatus for 30 min. The gels were rinsed with ultrapure water, then stained with approximately 10 mL of TMB, one component HRP membrane, for three hours, shaking at room temperature (RT). The gel was then de-stained by rinsing with ultrapure water and shaking overnight at RT. Images were captured using Syngene G: Box mini gel system wet scan at 647 nm and processed using the GenSys software.

Preservative effectiveness and bacterial endotoxin testing: These tests were conducted by PACE Analytical Life Sciences. Two lead liquid formulations containing 2PE (2-phenoxyethanol) preservative were tested according to USP 51. Lead lyophilized formulations were submitted for kinetic endotoxin testing according to USP 85 compendia.

### S-antigen bulk and formulation excipients

Bulk NDV-S-HXP S-antigen lots manufactured in 2021 were kindly provided by the Institute of Butantan (Lots 200004, 200005, 210001, 210002, and 210003). Each vaccine lot was tested via the S-antigen inhibition ELISA prior to use. A final S-antigen concentration of 6  µg/mL (3  µg dose) was selected for use in this study based on results from the clinical Phase I studies conducted in Vietnam and Thailand. [4], [5].

Interference of each of the individual excipients (shown in Table 1) with the S-antigen ELISA were evaluated by mixing each excipient with S-antigen to a final concentration of 6  µg/mL and testing by ELISA (n = 3). After initial excipient screening, a one-week stability study was conducted to further evaluate each excipient and preservative candidate at 2 °C to 8 °C and 40 **°**C by ELISA. Lead excipients and preservatives were selected based on S-antigen compatibility, availability of the excipient in LMIC settings, cost of the excipient, and use in existing commercial vaccine formulations.

**Table 1:** List of materials, reagents, and supplies.

Materials, reagents, and supplies	Vendor	Catalog number
Sucrose	JT Baker	4074–01
Histidine	JT Baker	2080–05
Glycine	JT Baker	4059–02
Arginine	Sigma	A1271000
Mannitol	JT Baker	25311.297
Sorbitol	Sigma	1,617,000
Polysorbate 80 (PS80)	Spectrum	PO138
Hydrolyzed gelatin	Sigma	G0262
2-Phenoxyethanol	Sigma	77,699
Lactalbumin hydrolysate	Spectrum	L3065
Polyvinylpyrrolidone (PVP)	Spectrum	P1416
Trehalose	Spectrum	T9601
Thimerosal	Spectrum	TH125
Sodium chloride	Sigma	S9888
Potassium chloride	Sigma	P3911
Sodium phosphate dibasic	Sigma	S9763
Potassium phosphate monobasic	Sigma	P0662
Combicoulomat fritless	Sigma	1,092,570,500
2-mL glass vials	Wheaton	223,683
5-mL glass vials	Wheaton	223,685
Vial stoppers	Wheaton	W224100-202
Lyophilization vial stoppers	Wheaton	W010938J
Aluminum crimp cap 5-mL vial	Wheaton	73822B-20
Aluminum crimp cap 2-mL vial	Wheaton	7-3822A-13
1X bolt sample buffer	Invitrogen	LC6678
Precision plus protein all blue	Bio-Rad	1610373EDU
TMB	Bio FX	ESPM-1000–01
15-well premade gel	Invitrogen	NW04120BOX
Anhydrous methanol	Sigma	34,860

Formulations were prepared with down selected excipients at the concentrations shown in Table 3. These were 0.25 µm sterile filtered and mixed with NDV-S-HXP antigen to achieve a 1 mL mixture at a target S-antigen concentration of 6 µg/mL. Once mixed, formulations were filled in 2-mL presterilized glass vials prior to testing by ELISA. Two preservative candidates (2PE at 5 mg/1 mL dose and 0.01 percent thimerosal) were included. A presterilized rubber stopper and aluminum crimp cap was placed on each vial and sealed with an automatic crimper prior to storage. Each test formulation was prepared in duplicate. NDV-HXP-S control samples were diluted to 6  µg/mL in phosphate-buffered saline (PBS) and held at −80 °C prior to testing.

### Formulation development

Seven liquid and seven lyophilized formulations were subjected to an accelerated temperature stability study at 40 °C and 2 °C to 8 °C for four weeks and S-antigen potency was evaluated by ELISA. Bulk S-antigen was mixed with PBS, selected excipients, and preservative in liquid formulations to achieve a final S-antigen concentration of 6 µg/mL. Each test formulation was aliquoted at 1-mL volumes into 2-mL glass vials. Lyophilized formulations were also prepared with selected excipients and filled into glass vials prior to lyophilization. Duplicate test vials were prepared and tested for each formulation at each test temperature and time point.

### Lyophilization cycle

Since multiple formulations needed to be lyophilized at the same time, a very conservative cycle was used for the lyophilization, as shown in Table 2. Briefly, samples were quickly cooled to −40 °C and maintained at −40 °C for 120 min, then temperature was gradually increased to −10 °C and performed annealing at −10 °C, then cooled to −40 °C again. The vacuum was applied to initiate primary drying. Temperature slowly increased to –33 °C, −28 °C, −10 °C, and 0 °C and dried at each temperature. Temperature gradually increased to 30 °C for 450 min. The temperature was cooled to 4 °C for storage after finishing secondary drying.

**Table 2:** Lyophilization cycle conditions.

Steps	Shelf temp (°C)	Ramp (min)	Hold (min)	Vacuum (mTorr)
Freeze	−40	0	120	
	−10	120	180	
	−40	120	300	
Primary drying	–33	90	240	100
	−28	120	900	
	−10	60	360	
	0	90	360	
Secondary drying	30	90	450	
Storage	4	60		
	4 (°C)

**Table 3:** Composition of the liquid and lyophilized multidose NDV-HXP-S formulations.

Formulation		Buffer	Excipient 1	Excipient 2	Excipient 3	Excipient 4	Excipient 5
LIQUID	F1	10 mM Phosphate + 137 mM saline	Sucrose	0.05 % PS80	−	−	2PE 2.5 mg/0.5 mL dose
	F2	10 mM Phosphate + 137 mM saline	Sucrose	0.05 % PS80	Gelatin	−	2PE 2.5 mg/0.5 mL dose
	**F3**	**10 mM Phosphate + 137 mM saline**	**Sucrose**	**0.05 % PS80**	−	**Arginine**	**2PE 2.5 mg/0.5 mL dose**
	F4	10 mM Phosphate + 137 mM saline	Sucrose	0.05 % PS80	Gelatin	Arginine	2PE 2.5 mg/0.5 mL dose
	**F5**	**10 mM Histidine + 137 mM saline**	**Sucrose**	**0.05 % PS80**	−	−	**2PE 2.5 mg/0.5 mL dose**
	**F6**	**10 mM Histidine + 137 mM saline**	**Sucrose**	**0.05 % PS80**	**Gelatin**	−	**2PE 2.5 mg/0.5 mL dose**
	F7	PBS 0.01 % Thimerosal	−	0.05 % PS80	−	−	−
LYO	F1	10 mM Phosphate + 137 mM saline	Sucrose	0.05 % PS80	Gelatin	Mannitol	−
	F2	10 mM Phosphate + 137 mM saline	Sucrose	0.05 % PS80	Gelatin	PVP	−
	**F3**	**10 mM Phosphate + 137 mM saline**	**Sucrose**	**0.05 % PS80**	**Gelatin**	**Mannitol**	**Histidine**
	F4	10 mM Phosphate + 137 mM saline	Sucrose	0.05 % PS80	Gelatin	Mannitol	Arginine
	**F5**	10 mM Phosphate + 137 mM saline	**Sucrose**	**0.05 % PS80**	**Gelatin**	**PVP**	**Glycine**
	F6	10 mM Phosphate + 137 mM saline	Sucrose	0.05 % PS80	Gelatin	Mannitol	Lactalbumin Hydrolysate
	F7	10 mM Phosphate + 137 mM saline	Sucrose	Potassium Glutamate	Gelatin	PVP	Lactalbumin Hydrolysate

### Six-month stability study

Three bulk S-antigen lots (21001–21003) were combined, then mixed with PBS, selected excipients, and preservative to achieve a final 2-mL mixture at a target concentration of 6 µg/mL, which was filled into 5-mL vials. Due to long lead times of presterilized stoppers and vials used in the previous study, 5-mL vials and stoppers were purchased unsterilized and had to be sterilized prior to use. We sterilized 5-mL vials by placing them into stainless steel trays, rinsing with ultrapure water, covering with the lid, and inverting to drain. This was repeated three times to rinse out the vials. The trays were then wrapped in a double-layer of aluminum foil and placed into oven set to 270 °C to dry for two hours before being left overnight to cool at 50 °C. Stoppers were autoclaved for 20 min at 121 °C in autoclave bags, with 100 stoppers per bag.

Vials were topped with sterile stoppers and sealed in the same method as previously described. Vials were stored at 40 °C, 25 °C, and 2 °C to 8 °C. Ambient humidity was used for all testing conditions. Each formulation candidate had three vials prepared for each temperature and tested for S-antigen content and pH, as well as observance of cake shrinkage and moisture content for lyophilized formulations, and visual assessment of particulates for liquid formats. Testing was repeated every month for the duration of the 6-month study.

## Results

**Fig. 1:**
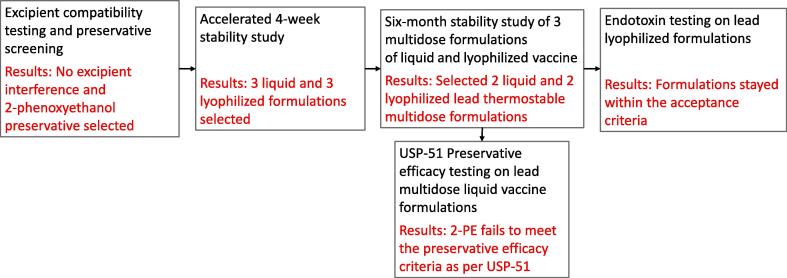
**NDV-HXP-S formulation development approach.** Schematic showing the liquid and lyophilization formulation development approach.

The approach to developing liquid and lyophilized formulations of the NDV-HXP-S vaccine are shown in Fig. 1**.** The inhibition ELISA as described in previously published methods to quantify the amount of S-antigen present in NDV-HXP-S formulations was used to evaluate the stability of the key antigen (spike) present in the NDV-HXP-S vaccine during formulation studies. [7] The antibody used in this method is specific to the spike protein of the Wuhan Variant as this is the target of the vaccine begin developed.

**Fig. 2:**
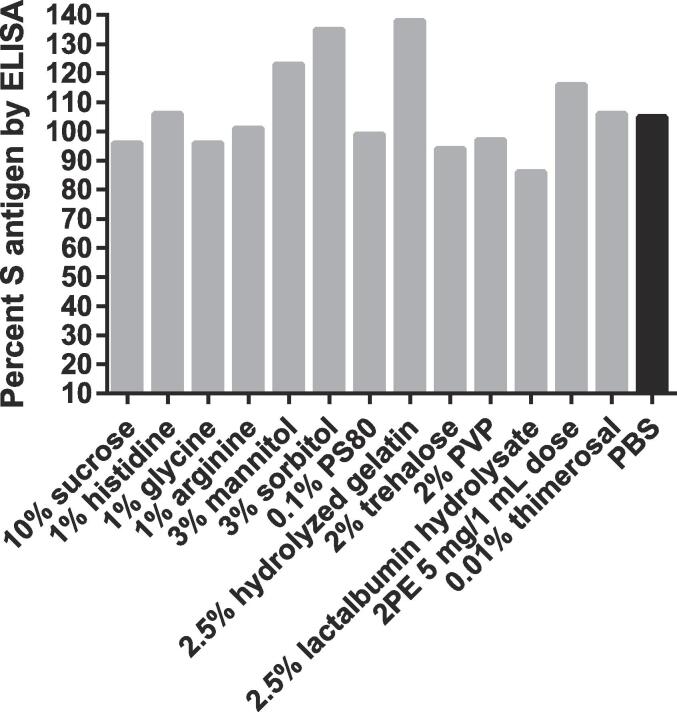
**Excipient compatibility screening**. Stability of the S-antigen with each of the excipients after holding for one week at 40 °C. (n = 2).

All proposed excipients shown in Table 1 were evaluated for interference in the S-antigen ELISA by formulating each excipient individually with S-antigen to a final relevant clinical concentration of 6 µg/mL. None of the excipients tested interfered with measuring the S-antigen content by ELISA at the concentrations tested. Excipients and preservatives were further down selected based on S-antigen stability after holding at 40 °C for one week and testing by ELISA (Fig. 2). S-antigen content was maintained with each of the excipients within the 20 % assay variability of the S-antigen ELISA after one week at 40 °C.

**Fig. 3:**
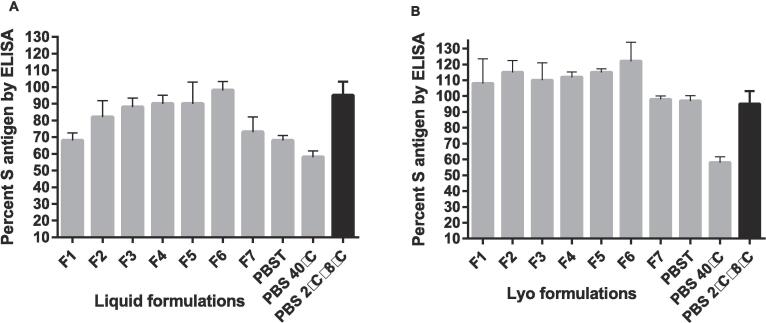
**Accelerated stability study of liquid and lyophilized formulations.** A. S-antigen content was evaluated in seven liquid formulation candidates after holding for four weeks at 40 °C. B. S-antigen content was evaluated in seven lyophilized formulation candidates after holding for four weeks at 40 °C (n = 2).

Following excipient screening, multiple liquid and lyophilized formulations were prepared for accelerated stability testing at 40 °C and 2 °C to 8 °C for four weeks, as shown in Table 3. Lead excipients and preservatives were selected based on S-antigen compatibility, availability of the excipient in LMIC settings, cost of excipient, and use in existing commercial vaccine formulations. Liquid and Lyophilized formulations were tested for S-antigen content by ELISA at 0, 1-, 2-, and 4-week time points Fig. 3**,** Figs. S1 and S2**.** pH, moisture content and visual appearance were tested at 0, 1-, 2-, and 4-week time points. All pH measurements for the formulations were approximately pH 7 and no visible particulates were observed. Lyophilized formulations had good cake appearances with no evidence of shrinkage over the 4-week study period (data not shown).

### Six-month stability study

**Fig. 4:**
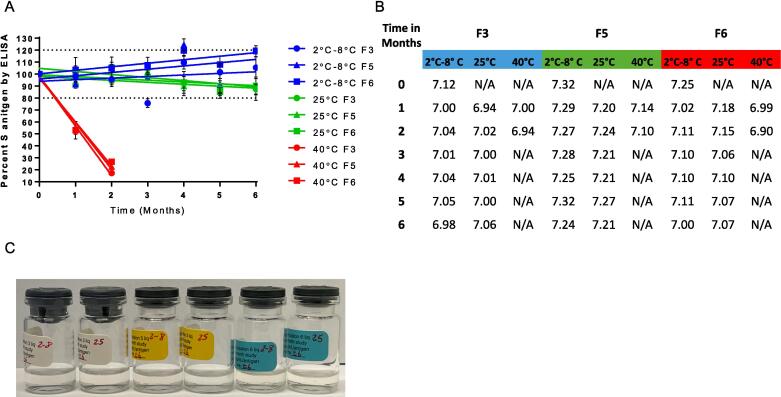
**Liquid formulation 6-month stability study**. Characterization of each of the three liquid formulation candidates after holding at 2 °C to 8 °C (blue), 25 °C (green) and 40 °C (red). Each data point represents n = 3 measurements. A. Percent of S-antigen recovered by ELISA at each monthly timepoint compared to the initial timepoint. B. Results of pH measurements at each monthly timepoint. C. Representational image of liquid formulation candidates from the 6-month time point.

A total of three liquid and three lyophilized formulations were selected for an extended 6-month stability study at 2 °C to 8 °C, 25 °C, and 40 °C based on the testing results from the accelerated 4-week stability study. Liquid formulations 3, 5, and 6 were selected and lyophilized formulations 3, 5, and 3 without protein stabilizer. Three vials of each formulation candidate were prepared for each temperature. All vials were evaluated by S-antigen potency by ELISA, visual appearance, and pH, with the additional observations of cake shrinkage and moisture content for lyophilized formulations. Liquid formulations were tested monthly throughout the 6-month stability study and results are shown in Fig. 4**.**

**Fig. 5:**
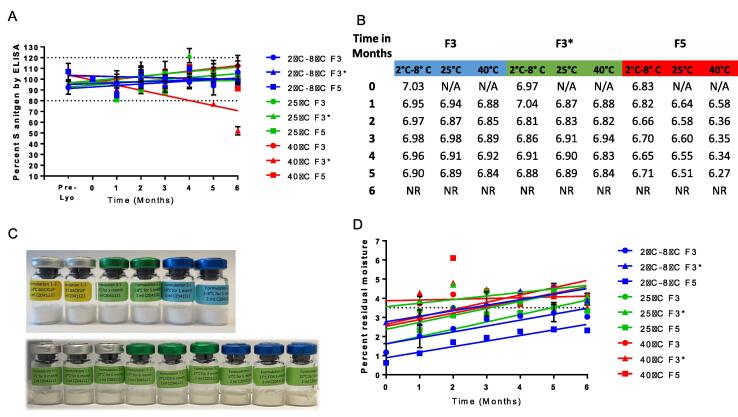
**Lyophilized formulation 6-month stability study**. Characterization of each of the three lyophilized formulation candidates after holding at 2 °C to 8 °C (blue), 25 °C (green) and 40 °C (red). Each data point represents n = 3 measurements. A. Percent of S-antigen recovered by ELISA at each monthly timepoint compared to the initial timepoint. B. Results of pH measurements at each monthly timepoint. 6-month pH time point was not recorded. C. Representational image of liquid formulation candidates from the 6-month time point. D. Results of the moisture content of each formulation at each monthly timepoint.

S-antigen content was stable for the 6-month study period at 2 °C to 8 °C and 25 °C in all liquid formulations tested. S-antigen content also remained stable in liquid formulations for up to two months at 40 °C (Fig. 4**A**). Lyophilized formulations were also tested monthly throughout the 6-month stability study and results are shown in Fig. 5. S-antigen remained stable at all test temperatures for the lyophilized formulations except for formulation F3* (formulation 3 without protein stabilizer) which showed decreased stability at the 6-month time point (Fig. 5**A**). Lyophilized formulations 3 and 5 had good cake appearances with no evidence of shrinkage, but we observed some shrinkage in formulation 3* at 40 °C (Fig. 5**C**).

**Fig. 6:**
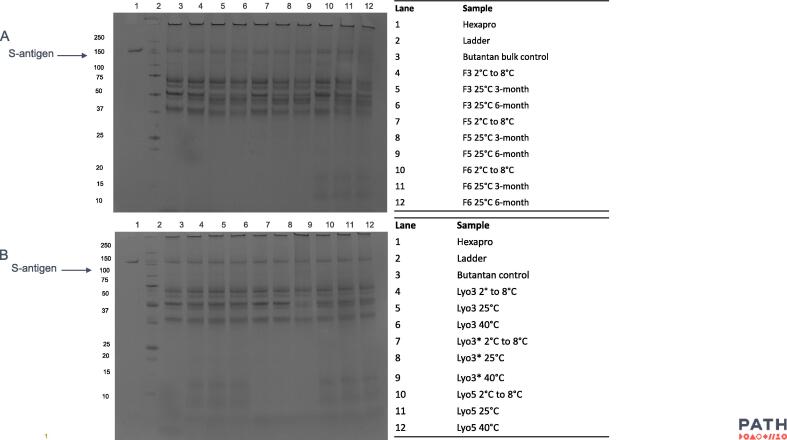
**S-antigen integrity by SDS-PAGE.** SDS-PAGE analysis was conducted on each of the liquid (A) and lyophilized (B) formulations from the 6-month stability study comparing 0, 3-, 6-month timepoints to the purified S-antigen Hexapro control.

No significant differences in pH or visible particles were observed for either liquid or lyophilized formulations for the duration of the 6-month stability study. The integrity of the S-antigen in each of the formulations was further evaluated by SDS-PAGE at the end of the stability study (Fig. 6). No differences were noted in the size or relative quantity of the S-antigen content in any of the formulations tested.

Preservative testing was performed on the two lead liquid formulations (3 and 6) stored at 2 °C to 8 °C. Table S1 outlines the acceptance criteria for each of the challenge organisms according to the USP 51 testing method. Based on the results of the preservative effectiveness testing, 2PE is not an effective preservative in the tested formulations for the NDV-HXP-S vaccine.

Endotoxin testing was performed on the two lead lyophilized formulations (3 and 5) stored at 25 °C and 40 °C according to USP 85. The results of the testing showed endotoxin levels less than 5 EU/mL which is within the USP 85 assay acceptance criteria for parenteral products (shown in Table 4).

**Table 4:** Two lead lyophilized formulations were selected for endotoxin testing according to USP method 85.

Sample	Result	Pass? (Yes/No)
Formulation 1 25 °C	3.3988 EU/mL	Yes
Formulation 3 25 °C	3.0275 EU/mL	Yes
Formulation 1 40 °C	2.6917 EU/mL	Yes
Formulation 3 40 °C	2.0895 EU/mL	Yes

## Discussion

When it comes to global distribution of COVID-19 vaccines, there is lack of a sustainable vaccine supply in LMICs, a result of several barriers, including manufacturing costs and cold chain delivery [1], [2].

To address manufacturing costs, the proposed vaccine candidate uses the same technology for vaccine production in eggs as that used in producing inactivated influenza vaccines. This approach is well-established and used worldwide for manufacturing more than a billion doses annually of influenza vaccine. The manufacturing considerations for NDV-HXP-S vaccine are captured in a separate publication and will not be discussed here [7]. Thermostability of NDV-HXP-S is another attribute that is critical for vaccines products intended for use in LMIC settings.

The NDV-HXP-S vaccine construct presents a novel platform, expressing pre-fusion stabilized S-protein (HexaPro) in a membrane-bound trimeric conformation [8], [9], [10]. The spike protein is found on the surface of SARS-CoV-2, the virus that causes COVID-19, and is responsible for its entry into host cells. [3], [11] The stability of the spike protein refers to how well it maintains its shape and function under different conditions, such as temperature, pH, or mutations. The stability of the spike protein can affect the infectivity of the virus, the immune response of the host, and the development of vaccines and therapeutics. Because of this function, this protein has been the target focus of most COVID-19 vaccines, including the Pfizer/BioNTech and Moderna mRNA vaccines. [13].

In this reported work, the efforts on developing a thermotolerant formulation were focused on maintaining the stability or conformation of the spike protein under different storage temperatures by developing formulations comprising a combination of stabilizing excipients. A stable liquid vaccine formulation is considered to be desirable due to multiple reasons (including ease of administration and potentially lower costs). However, they are more susceptible to degradation induced by pH changes, light, heat, and microbial growth. An alternative would be a lyophilized vaccine which has better stability, although it requires a preparation step for vaccine reconstitution with a vaccine-specific diluent prior to administration.

In this work, we used a two-pronged approach, where our strategy was to develop a formulation, composed of excipients that impart stability to the NDV-HXP-S vaccine and could be adapted for both liquid and lyophilized presentations with little modification to the composition. The GRAS (generally regarded as safe) excipients are were selected based on prior use in commercial vaccine products with an emphasis on minimizing the number of excipients and choosing those which are more widely available to keep the overall cost low for the vaccine formulation. [14] The base excipients which were common in both liquid and lyophilized formulations were sucrose and PS80. Additional excipients were added to make the formulation fit-for-purpose for lyophilization. Other modifications pertained to adding or withholding gelatin or amino acids. Sucrose is a commonly used excipient, known to provide protein stability in solutions. It influences the thermodynamics and kinetics of protein folding and unfolding and preserves the protein structure and function by forming a rigid and amorphous matrix that immobilizes the protein and prevents its degradation during the freeze-drying process. Glycine and arginine have different mechanisms of action for protein stability, but they both contribute to the proper folding and function of proteins. Glycine stabilizes proteins by preventing pH decrease in solutions and maintaining the proteins in an amorphous state. Arginine is positively charged at physiological pH and stabilizes proteins by forming electrostatic interactions with other charged groups on the protein surface or in the solvent. At the end of the four-week stability study for formulation down-selection, there was no discernable difference between the formulations (within the liquid and lyophilized) based on the criteria set for down selection. Therefore, for the six-month stability study, we downselected the candidates based on their composition, where, having fewer components would simplify the process of preparation and potentially the overall cost of the product. The results of the six-month stability evaluation showed that within the liquid NDV-HXP-S formulations, all three candidates (F3, F5, and F6) performed similarly in terms of physicochemical stability and maintaining the S-antigen content and integrity at 2 °C to 8 °C and 25 °C storage for up to six months. However, none of the liquid formulations were stable at 40 °C, where a significant drop in antigen content was observed within a week. Among the lyophilized formulations, two formulation candidates, F3 and F5 were found to be most stable in maintaining all physicochemical properties and the S-antigen content and antigen integrity at all storage temperatures (2 °C to 8 °C, 25 °C, and 40 °C) at the end of six-month stability study. Lyophilized formulation F3*, despite having no protein stabilizer, maintained the antigen stability at 2 °C to 8 °C and 25 °C storage. However, unlike other lyophilized formulations, this candidate could not maintain the S-antigen content at 40 °C storage, suggesting the role of gelatin in imparting stability to the protein as previously reported by several groups. [15], [16] The moisture content of a lyophilized vaccine is a critical attribute and is a part of the release specification for the product, as it can significantly impact the stability of the vaccine. For lyophilized vaccines, the conventional approach is to keep the desired moisture specification as low as possible, typically under 3 %, however this specification is highly dependent on the stability profile of the vaccine antigen under the targeted storage condition. At the end of the six-month stability study, the moisture content, increased from initial (T0) timepoint, however, using the stability indicating assay for NDV-HXP-S, it did not appear to impact the stability of the S-antigen, especially at 2-8C and 25C storage conditions for the three lyophilized formulations. It was surprising to see that a similar stabilizing effect was not seen in the liquid formulations stored at 40 °C.

Since improving the temperature stability of COVID-19 vaccines is expected to improve storage, distribution, and administration costs, further reduction in cost can be achieved with a multidose presentation of the vaccine. [17] According to the CDC, in general, a preservative is needed for a liquid multidose vaccine to prevent microbial growth in the event that the vaccine is accidentally contaminated, as might occur with repeated puncture of multidose vials with a needle. Preservatives can kill or prevent the growth of microorganisms, particularly bacteria and fungi. [18] Both thimerosal and 2PE are commonly used preservatives found in marketed liquid multidose vaccines, and these were used at concentrations based on those reported in the marketed vaccines. [19] In our study, out of the two preservatives tested, 2PE was shown to be compatible with the antigen, where the S-antigen content was maintained throughout the stability study based on ELISA results. Thimerosal showed incompatibility with the antigen and was eliminated early on in the formulation development stage. Once the 2PE preservative compatibility was established, the next step was to evaluate preservative effectiveness. The preservative effectiveness test is a standard method to determine the ability of a preservative to inhibit the growth of microorganisms in a vaccine formulation. USP 51 is the compendial method which describes the procedure and criteria for testing the ability of a preservative to inhibit the growth of microorganisms in products that are packaged in multidose presentations. [20] After the completion of the six-month stability study, two liquid multidose formulations (F3 and F6) containing 2PE were tested for preservative effectiveness following the USP 51 test procedure. As per the USP 51 criteria for parenteral products, the effective preservative should be able to prevent microbial growth of all five test organisms at the end of day 14. Our test results showed that 2PE was ineffective at preventing the growth of all three bacterial test organisms and the fungi *Candida Albicans* by day 7. At this concentration, 2PE was only able to prevent the fungal growth of the *Aspergillus brasiliensis* in the tested formulations. Since the preservative did not meet the day 7 criterion for all five test organisms, the testing was discontinued. It is unclear why the USP 51 testing failed for 2PE considering that the levels are similar to those reported in the marketed vaccine products. The formulation excipients sucrose, arginine, gelatin are common vaccine excipients; it is unlikely that these were neutralizing the effectiveness of 2PE. The other explanation could be if the presence of the surfactant PS80 somehow renders 2PE ineffective by trapping this hydrophobic preservative within the micelles, making it unavailable for preventing the microbial growth of the test organism. PS80 is an essential component of the NDV-HXP-S formulation and is commonly added to vaccine formulations to help prevent the aggregation of viral particles in solution. If 2PE must be included in multidose formulations, the interference due to PS80 needs to be confirmed by testing the preservative effectiveness of 2PE at various concentrations while ensuring that the levels tested are not detrimental to the antigen and also do not compromise safety. Another option would be pursuing a single-use multidose format for this vaccine, containing no preservatives, where opened multidose vaccine vials should be discarded at the end of the immunization session, or within six hours after opening, whichever comes first. [21].

Study Limitation: In this study, the liquid and lyophilized formulations were developed using excipients commonly used in existing commercial vaccines and considered as inactive ingredients in the FDA database. They are expected to have minimal to no impact on immunogenicity based on their prior use with vaccines and other injectable products. The impact of the various excipients on the immunogenicity and protective efficacy of the NDV-HXP-S immunogen was outside the scope of the current work and was not addressed in this study.

## Conclusion

We were able to overcome the stability challenges with currently marketed COVID-19 vaccines which require cold storage (2 °C to 8 °C) or ultracold temperatures (–20 °C or –80 °C) for long-term storage. The freeze-dried formulations maintained the antigen (spike protein) stability even after continuous exposure to 40 °C temperatures for up to six months (the longest time point tested). This stability attribute aligns with the American Pandemic Preparedness plan where the developed thermotolerant vaccine formulations could be stockpiled and transported to various outreach areas which will improve vaccine equity and coverage.

## CRediT authorship contribution statement

**Anan Bzami:** Writing – original draft, Visualization, Investigation, Formal analysis, Data curation. **Changcheng Zhu:** Writing – original draft, Validation, Methodology, Data curation. **Marcus Estrada:** Writing – original draft, Methodology, Investigation, Formal analysis, Data curation, Conceptualization. **Jessica A. White:** Writing – review & editing, Supervision, Project administration, Formal analysis, Data curation. **Manjari Lal:** Writing – review & editing, Writing – original draft, Project administration.

## Declaration of competing interest

The authors declare that they have no known competing financial interests or personal relationships that could have appeared to influence the work reported in this paper.

## Data Availability

Data has been attached in Files section.

## References

[1] Kumru O.S. (2014). Vaccine instability in the cold chain: Mechanisms, analysis and formulation strategies. Biol J Int Assoc Biol Stand.

[2] Fahrni M.L. (2022). Management of COVID-19 vaccines cold chain logistics: A scoping review. J Pharm Policy Pract.

[3] Sun W. (2021). A Newcastle disease virus expressing a stabilized spike protein of SARS-CoV-2 induces protective immune responses. Nat Commun.

[4] Duc Dang A. (2022). Safety and immunogenicity of an egg-based inactivated Newcastle disease virus vaccine expressing SARS-CoV-2 spike: Interim results of a randomized, placebo-controlled, phase 1/2 trial in Vietnam. Vaccine.

[5] Pitisuttithum P. (2022). Safety and immunogenicity of an inactivated recombinant Newcastle disease virus vaccine expressing SARS-CoV-2 spike: Interim results of a randomised, placebo-controlled, phase 1 trial. eClinicalMedicine.

[6] Governmental Pharmaceutical Organization. (2024, January 15). GPO announced an emergency use authorization of the COVID-19 vaccine as a booster dose after a successful clinical trial phase 3 [Press release]. https://www.gpo.or.th/view/1029.

[7] Estrada, M et. al. Development of a quantitative ELISA for SARS-CoV-2 vaccine candidate, NDV-HXP-S, with CpG 1018® adjuvant. *Human Vaccines and Immunotherapeutics (in press)*. 10.1080/21645515.2024.2315709 PMC10877971 38372198

[8] Hsieh C.-L., McLellan J.S. (2022). Protein engineering responses to the COVID-19 pandemic. Curr Opin Struct Biol.

[9] Carreño J.M. (2023). An inactivated NDV-HXP-S COVID-19 vaccine elicits a higher proportion of neutralizing antibodies in humans than mRNA vaccination. Sci Transl Med.

[10] Tcheou J. (2021). Safety and immunogenicity analysis of a newcastle disease virus (NDV-HXP-S) expressing the spike protein of SARS-CoV-2 in Sprague Dawley rats. Front Immunol.

[11] Sun W. (2020). A Newcastle Disease Virus (NDV) expressing a membrane-anchored spike as a cost-effective inactivated SARS-CoV-2 vaccine. Vaccines.

[12] Drain P.K., Nelson C.M., Lloyd J.S. (2003). Single-dose versus multi-dose vaccine vials for immunization programmes in developing countries. Bull World Health Organ.

[13] Barrett C.T. (2021). Effect of clinical isolate or cleavage site mutations in the SARS-CoV-2 spike protein on protein stability, cleavage, and cell–cell fusion. J Biol Chem.

[14] U.S. Food and Drug Administration. Vaccine Excipient Summary. (2021).

[15] Kumru O.S. (2019). Stabilization and formulation of a recombinant Human Cytomegalovirus vector for use as a candidate HIV-1 vaccine. Vaccine.

[16] Eilts F. (2023). An investigation of excipients for a stable Orf viral vector formulation. Virus Res.

[17] Basu S., Rustagi R. (2022). Multi-dose vials versus single-dose vials for vaccination: Perspectives from lower-middle income countries. Hum Vaccines Immunother.

[18] CDC. Questions about Multi-dose vials. (2019).

[19] Institute for Vaccine Safety. Excipients in Routinely Recommended Vaccines. (2023).

[20] Onaghise, O. Pharmaceutical Microbiology Manual. (2020).

[21] WHO. WHO Policy Statement: Multi-dose Vial Policy (MDVP). (2014).

