# Supplementary material for: Development of multidose thermotolerant formulations of a vector-based Covid-19 vaccine candidate, NDV-HXP-S in different product formats: Stability and preservative efficacy study
Source: Vaccine X. 2024 Jul 27;20:100535. doi: 10.1016/j.jvacx.2024.100535 (PMC11345403; doi:10.1016/j.jvacx.2024.100535)
Supplement: Supplementary Data 1 [file mmc1.docx]

**Table S1. USP 51 acceptance criteria and lead liquid formulations results**

| Test Organisms | Log10 initial population | Log10 reduction Formulation 3  2ºC-8ºC  Day 7 | Log10 reduction Formulation 6  2ºC-8ºC  Day 7 | LM 171 logic acceptance criterion | Pass? (Yes/No) |
| --- | --- | --- | --- | --- | --- |
| Pseudomonas aeruginosa | 5.6 | -2.7 | > -2.8 | ≥ 1.0 | No |
| Escherichia coli | 5.7 | -2.3 | > -2.7 | ≥ 1.0 | No |
| Staphylococcus aureus | 5.7 | 0.2 | > -2.7 | ≥ 1.0 | No |
| Candida albicans | 5.4 | -1.1 | 1.9 | No increase | No |
| Aspergillus brasiliensis | 5.6 | -0.2 | 0.2 | No increase | Yes |
